# Rare and New Lung Diseases

**Published:** 1918-08

**Authors:** 


**Rare and New Lung Diseases.** The Maryland Med. Jour., Jan. 1918, editorially calls attention to the following: Fibrous Bronchiolitis, first recognized 1901, showing greyish white nodules resembling military tubercles on section, the same casting shadows by X-rays, this disease following irritating gases, measles and pertussia. Principal symptoms dyspnoea and cyanosis. Pulmonary Blastomycosis not recognized much before 1914, resembling acute tuberculosis in onset and signs, but microscopically negative as to tubercle bacilli and often positive as to blastomyces. KI and tonics may help. Coccidioid Granuloma, known in California for about 21 years, usually begins like pulmonary tuberculosis, later developing skin lesions. All pulmonary cases thus far observed have been fatal. Pulmonary Aspergillosis, known for about 20 years, begins like bronchitis, with cough, sputum soon becomes purulent and blood-streaked, indigestion, night sweats, evening fever, emaciation, bronchial dilatation. Haemoptysis may be the initial symptom. It may be relieved spontaneously, leaving induration. KI and as advised. Sporotrichosis may simulate tuberculosis, as to cough, sputum, apical dullness and rales. Differentiation by identification of fungus. Organism thrives on some vegetables and has apparently been conveyed by handling coffee. Chiefly reported from Mississippi Valley. Streptotrichosis strongly resembles tuberculosis. Organisms frequent on food stuffs and may lodge in tonsillar crypts. It may invade a single apex and gradually spread to other parts of the lungs. Diastomiasis occurs in Asiatics, who may live 30 years with this disease or become spontaneously cured. Thus far, it has not been reported in native Americans or other foreigners. Actinomyeosis, long known and well described but liable to be confused with tuberculosis. Never diagnose a pulmonary infection without examination of sputum—and, we may add, the examination should be repeated sufficiently often.

